# Supplementary material for: A Cyclen-Functionalized Cobalt-Substituted Sandwich-Type Tungstoarsenate with Versatility in Removal of Methylene Blue and Anti-ROS-Sensitive Tumor Cells
Source: Molecules. 2022 Sep 30;27(19):6451. doi: 10.3390/molecules27196451 (PMC9573041; doi:10.3390/molecules27196451)
Supplement: Supplementary file 1 [file molecules-27-06451-s001.zip › molecules-1871576-supplementary.pdf]

molecules MDPI

Supplementary File

A Cyclen-Functionalized Cobalt-Substituted Sandwich-Type Tungstoarsenate with Versatility in Removal of Methylene Blue and Anti-ROS-Sensitive Tumor Cells

Jiai Hua [1,2,†], Xueman Wei [3,†], Yifeng Li [1], Lingzhi Li [2], Hui Zhang [1], Feng Wang [2,*], Changli Zhang [4,*] and Xiang Ma [1,5,*]

[1] Chemistry and Chemical Engineering Department, Taiyuan Institute of Technology, Taiyuan, 030008, China
[2] Laboratory of Biochemistry and Pharmacy, Taiyuan Institute of Technology, Taiyuan, 030008, China
[3] Department of Geriatrics, First affiliated Hospital of Naval Medical University, Shanghai, 200081, China
[4] School of Environmental Science, Nanjing Xiaozhuang University, Nanjing, 2111171, China
[5] State Key Laboratory of Coordination Chemistry, School of Chemistry and Chemical Engineering, Nanjing University, Nanjing 210023, China
* Correspondence: wangfeng00468@163.com (F.W.); zhangcl@njxzc.edu.cn (C.Z.); maxiang@tit.edu.cn (X.M.); Tel.: +86-351-356-9476 (X.M.)
† These authors contributed equally to this work.

Table S1. Bond valence and Σs of W, Co, As in CAW.

Bond	Valence	Bond	Valence	Bond	Valence	Atom	Σs
W(1)-O(5)	1.708	W(1)-O(6)	2.366	W(1)-O(11)	1.944		
W(1)-O(15)	1.93	W(1)-O(22)	2.027	W(1)-O(27)	1.755	W1	6.046
W(2)-O(1)	2.383	W(2)-O(7)	1.767	W(2)-O(13)	1.712		
W(2)-O(17)	2.049	W(2)-O(20)	1.965	W(2)-O(24)	1.925	W2	5.903
W(3)-O(4)	1.911	W(3)-O(6)	2.481	W(3)-O(16)	1.962		
W(3)-O(22)	1.898	W(3)-O(30)	1.894	W(3)-O(34)	1.717	W3	5.779
W(4)-O(9)	1.970	W(4)-O(10)	1.834	W(4)-O(12)	1.950		
W(4)-O(16)	1.871	W(4)-O(31)	2.490	W(4)-O(33)	1.671	W4	6.137
W(5)-O(6)	2.369	W(5)-O(8)	1.829	W(5)-O(11)	1.928		
W(5)-O(26)	1.682	W(5)-O(29)	1.924	W(5)-O(30)	1.979	W5	6.605
W(6)-O(1)							
W(6)-O(9)	2.466	W(6)-O(3)	1.957	W(6)-O(4)	1.928		
	1.880	W(6)-O(17)	1.865	W(6)-O(19)	1.736	W6	5.807
W(7)-O(10)							
W(7)-O(28)	2.011	W(7)-O(23)	1.888	W(7)-O(25)	1.824		
	1.718	W(7)-O(29)	1.918	W(7)-O(31)	2.405	W7	5.941
W(8)-O(1)							
W(8)-O(18)	2.323	W(8)-O(3)	2.080	W(8)-O(15)	1.900		
W(9)-O(2)	1.754	W(8)-O(21)	1.935	W(8)-O(32)	1.760	W8	5.882
W(9)-O(23)							
As(1)-O(1)	1.766	W(9)-O(12)	2.056	W(9)-O(20)	1.716		
As(1)-O(31)	1.964	W(9)-O(24)	1.915	W(9)-O(31)	2.333	W9	5.944
Co(1)-O(8)	1.671	As(1)-O(6)	1.666	As(1)-O(14)	1.686		
Co(1)-O(25)	1.661					As1	5.186
Co(2)-O(2)	2.125	Co(1)-O(13)	2.062	Co(1)-O(14)	2.208		
Co(2)-O(14)	2.140	Co(1)-O(32)	2.079	Co(1)-O(1W)	2.145	Co1	1.869
Co(3)-O(3)							

Co(3)-N(3)	1.995	Co(2)-O(81)	2.132	Co(2)-O(14#)	2.187		
Co(4)-N(5)	2.188	Co(2)-O(25)	2.109	Co(2)-O(27#)	2.002	Co2	2.026
Co(4)-O(12)							
	1.999	Co(3)-N(1)	2.160	Co(3)-N(2)	2.130		
	2.100	Co(3)-N(4)	2.150			Co3	1.742
	2.095	Co(4)-N(6)	2.080	Co(4)-N(7)	2.060		
	1.998	Co(4)-N(8)	2.100			Co4	1.935

Table S2. Selected bond length (Å) for CAW.

W1-O5	1.708(12)	W5-O26	1.682(11)	As1-O1	1.671(10)
W1-O6	2.366(10)	W5-O29	1.924(11)	As1-O6	1.666(10)
W1-O11	1.944(10)	W5-O30	1.979(11)	As1-O14	1.686(10)
W1-O15	1.936(11)	W6-O1	2.466(10)	As1-O31	1.661(10)
W1-O22	2.027(10)	W6-O3	1.957(11)	Co1-O8	2.125(10)
W1-O27	1.755(10)	W6-O4	1.928(12)	Co1-O13	2.062(11)
W2-O1	2.383(10)	W6-O9	1.880(10)	Co1-O14	2.208(10)
W2-O7	1.712(12)	W6-O17	1.865(11)	Co1-O25	2.140(10)
W2-O13	1.767(11)	W6-O19	1.736(11)	Co1-O32	2.079(11)
W2-O17	2.049(11)	W7-O10	2.011(10)	Co1-O1W	2.145(13)
W2-O21	1.965(11)	W7-O23	1.888(11)	Co2-O2	1.995(10)
W2-O24	1.925(12)	W7-O25	1.824(10)	Co2-O8	2.132(10)
W3-O4	1.911(12)	W7-O28	1.718(10)	Co2-O14	2.187(10)
W3-O6	2.481(10)	W7-O29	1.918(11)	Co2-O14	2.188(11)
W3-O16	1.962(11)	W7-O31	2.405(10)	Co2-O25	2.109(11)
W3-O22	1.898(11)	W8-O1	2.323(10)	Co2-O27	2.002(10)
W3-O30	1.894(11)	W8-O3	2.080(10)	Co3-O3	1.999(11)
W3-O34	1.717(11)	W8-O15	1.900(11)	Co3-N1	2.16(2)
W4-O9	1.970(11)	W8-O18	1.754(12)	Co3-N2	2.13(2)
W4-O10	1.834(11)	W8-O21	1.935(11)	Co3-N3	2.10(2)
W4-O12	1.950(11)	W8-O32	1.760(10)	Co3-N4	2.15(2)
W4-O16	1.871(11)	W9-O2	1.766(10)	Co4-O12	1.998(11)
W4-O31	2.490(10)	W9-O12	2.056(11)	Co4-N5	2.095(19)
W4-O33	1.671(11)	W9-O20	1.716(10)	Co4-N6	2.080(15)
W5-O6	2.369(10)	W9-O23	1.964(11)	Co4-N7	2.06(2)
W5-O8	1.829(10)	W9-O24	1.915(11)	Co4-N8	2.100(18)
W5-O11	1.928(10)	W9-O31	2.333(10)		
